# Supplementary material for: Effect of dynamic taping on neck pain, disability, and quality of life in patients with chronic non-specific neck pain: a randomized sham-control trial
Source: PeerJ. 2024 Jan 26;12:e16799. doi: 10.7717/peerj.16799 (PMC10823991; doi:10.7717/peerj.16799)
Supplement: File S1 [file peerj-12-16799-s002.docx]

**Clinical Guidelines for Chronic Non-Specific Neck Pain – National Institute of Medical sciences, India**

The following activities are conducted alongside the taping sessions (both sham and experimental groups)

1. ***Pain Management Techniques: Modalities like transcutaneous electrical nerve stimulation (TENS)***

Traditional TENS (Low-Frequency TENS):

- *Frequency Range:* Typically ranges from 2 to 10 Hertz (Hz).
- *Pulse Duration:* Longer pulse durations.
- *Sensation:* Patients often feel a tingling or massaging sensation.
- *Sensory Threshold:* Set intensity just below the sensory threshold, ensuring nerve stimulation.

*Note: TENS was applied immediately after the tape was removed every 3^rd^ day.*

1. ***Stretching Exercises:***

A. Neck Stretch:

- Slowly tilt your head to one side, bringing your ear toward your shoulder.
- Hold for 15-30 seconds, feeling the stretch on the opposite side.
- Repeat on the other side.
- 2-3 repetitions on each side.

B. Neck Rotation:

- Gently turn your head to one side, looking over your shoulder.
- Hold for 15-30 seconds, feeling the stretch in the neck.
- Repeat on the other side.
- 2-3 repetitions on each side.

C. Neck Flexion and Extension:

- For flexion, lower your chin to your chest and hold for 15-30 seconds.
- For extension, tilt your head back, looking up toward the ceiling, and hold for 15-30 seconds.
- 2-3 repetitions of flexion and extension.

D. Shoulder Stretch:

- Bring your right arm across your chest.
- Use your left hand to gently pull your right arm closer.
- Hold for 15-30 seconds, feeling the stretch in your shoulder.
- Repeat on the other side.
- 2-3 repetitions on each side.

E. Upper Back Stretch:

- Clasp your hands in front, straighten your arms, and round your upper back.
- Hold for 15-30 seconds.
- Repeat 2-3 times.

F. Levator Scapulae Stretch:

- Tilt your head to the side and slightly forward.
- Place your hand on the opposite side of your head and apply gentle pressure.
- Hold for 15-30 seconds.
- Repeat on the other side.
- 2-3 repetitions on each side.

1. ***Therapeutic Exercises: Targeted exercises to strengthen the neck, shoulder, and upper back muscles.***

A. Neck Exercises:

- Neck Flexion and Extension: 2 sets of 10 repetitions.
- Neck Lateral Flexion: 2 sets of 10 repetitions on each side.
- Neck Rotation: 2 sets of 10 repetitions on each side.

B. Shoulder Exercises:

- Shoulder Blade Squeezes: 2 sets of 15 repetitions.
- Shoulder Circles: 1 set of 10 small circles, gradually increasing to 2 sets.
- Rotator Cuff Strengthening: 2 sets of 12 repetitions for each rotation.

C. Upper Back Exercises:

- Thoracic Extension: 2 sets of 12 repetitions.
- Lateral Pulldowns: 2 sets of 12 repetitions.
- Upper Back Rows: 2 sets of 12 repetitions.

1. ***Cervical Traction: Application of gentle traction to the cervical spine.***

- Patient Position: Supine B. Intensity: 1/7th of the body weight. C. Duration: 20 minutes

The following activities are communicated to the participants once at the beginning of the trial:

1. ***Education: Providing information on posture, ergonomics, and lifestyle modifications.***

Lifestyle Modifications:

- Discuss factors contributing to neck and shoulder pain.
- Provide strategies for movement and exercise.
- Emphasize staying hydrated and maintaining a healthy diet.

Ergonomic Workspace Setup:

- Ensure ergonomically designed workspaces.
- Adjust chair height, use a supportive chair, and position the monitor at eye level.

Frequent Breaks and Stretching:

- Encourage regular breaks during prolonged sitting.
- Incorporate neck stretches and movements during breaks.

Proper Phone Use:

- Avoid cradling the phone between ear and shoulder.
- Use hands-free devices for prolonged conversations.

Lifting Techniques:

- Promote proper lifting techniques.
- Bend at the knees, keep objects close, and avoid excessive neck twisting.

Limit Screen Time:

- Reduce time spent on electronic devices.
- Address poor neck posture associated with screen use.

Supportive Pillows and Mattress:

- Choose pillows and a mattress for optimal neck and spine support.
- Maintain a neutral spine position during sleep.

Avoiding Prolonged Static Positions:

- Discourage prolonged static positions.
- Encourage changes in posture and movement throughout the day.

1. *Home Exercise Program: Personalized exercise routine for continued therapy at home.*

A. Neck Flexion Stretch:

- Instructions: Sit or stand, lower chin toward chest.
- Repetitions: 3 sets of 15 seconds.
- Frequency: Daily.

B. Neck Extension Stretch:

- Instructions: Tilt head backward, looking up.
- Repetitions: 3 sets of 15 seconds.
- Frequency: Daily.

C. Neck Lateral Flexion Stretch:

- Instructions: Gently tilt head to the side, feeling stretch.
- Repetitions: 3 sets of 15 seconds on each side.
- Frequency: Daily.

D. Neck Rotation Stretch:

- Instructions: Turn head to the side, feeling a gentle twist.
- Repetitions: 3 sets of 15 seconds on each side.
- Frequency: Daily.

E. Shoulder Blade Squeezes:

- Instructions: Sit or stand, squeeze shoulder blades.
- Repetitions: 3 sets of 15.
- Frequency: Daily.

F. Chin Tucks:

- Instructions: Sit or stand with a straight spine, tuck chin.
- Repetitions: 3 sets of 15.
- Frequency: Daily.

G. Scapular Retraction:

- Instructions: Squeeze shoulder blades together.
- Repetitions: 3 sets of 15.
- Frequency: Daily.

H. Neck Isometric Exercises:

- Instructions: Press palm against forehead, resisting with neck muscles.
- Repetitions: 3 sets of 10.
- Frequency: Every other day.

I. Postural Correction Exercises:

- Instructions: Maintain neutral spine, perform shoulder rolls.
- Frequency: Throughout the day.

J. Aerobic Exercise:

- Instructions: Engage in low-impact aerobic activities.
- Duration: 20-30 minutes, 3 times a week.

Note: Adherence to the home program was self-reported.

Top of Form
