# Supplementary material for: Effect of dynamic taping on neck pain, disability, and quality of life in patients with chronic non-specific neck pain: a randomized sham-control trial
Source: PeerJ. 2024 Jan 26;12:e16799. doi: 10.7717/peerj.16799 (PMC10823991; doi:10.7717/peerj.16799)
Supplement: File S2 [file peerj-12-16799-s003.docx]

**SUPPLEMENTARY TABLES;**

Contents

[Table 1a Baseline characteristics of study participants including all the participants (with dropouts) those who were enrolled (n = 140) 2](#_Toc153696989)

[Table 1b; Gender wise difference analysis of main effect and interaction effect for the PROM's comparison at baseline and post-intervention 4 weeks. 3](#_Toc153696990)

[Table 1c; Occupation wise difference analysis of main effect and interaction effect for the PROM's comparison at baseline and post-intervention 4 weeks. 3](#_Toc153696991)

[Table 1d; Level of physical activity wise difference analysis of main effect and interaction effect for the PROM's comparison at baseline and post-intervention 4 weeks. 4](#_Toc153696992)

[Table 1e; Smoking habit (smoker versus non-smokers) wise difference analysis of main effect and interaction effect for the PROM's comparison at baseline and post-intervention 4 weeks. 4](#_Toc153696993)

# Table 1a Baseline characteristics of study participants including all the participants (with dropouts) those who were enrolled (n = 140)

|  | **Analyzed (n = 136)** | | | **Drop outs** (did not start or complete treatment) **n = 4** | |
| --- | --- | --- | --- | --- | --- |
| **Variables** | **DTC**  **(n = 69)** | **STC**  **(n = 67)** | **p value** | **DTC**  **(n = 1)** | **STC**  **(n = 3)** |
| Age (years, mean ± SD) | 42.74 ± 8.7 | 44.82 ± 7.43 | 0.137 | 44 | 41.6±9.2 |
| ^a^BMI kg/m^2^ | 25.97 ± 2.97 | 25.52 ± 3.24 | 0.40 | 24.0 | 28±1.1 |
| Chronicity of neck pain ( Mo) | 45.31 ± 19.37 | 43.4 ± 28.24 | 0.64 | 50 | 20.0±6.9 |
| ^a^Gender, n (%) |  |  |  |  |  |
| Male | 23 (33.3) | 29 (43.3) | **0.01** | 1(100) | 0(0) |
| Female | 46 (66.7) | 38 (56.7) |  | 0(0) | 3(100) |
| ^a^Marital status, n (%) |  |  |  |  |  |
| Married | 35 (50.7) | 40 (59.7) | 0.19 | 1(100) | 3(100) |
| Not married/single | 34 (49.3) | 27 (40.3) |  | 0(0) | 0(0) |
| ^a^Occupation, n (%) |  |  |  |  |  |
| Clerical/home maker | 14 (20.3) | 24 (35.8) | **0.009** | 0(0) | 0(0) |
| Business | 20 (29) | 06 (9) |  | 1(100) | 2(66.7) |
| Professionals | 14 (20.3) | 10 (14.9) |  | 0(0) | 1(33.3) |
| Skilled labor | 21 (30.4) | 27 (40.3) |  | 0(0) | 0(0) |
| ^a^Level of activity n (%) |  |  |  |  |  |
| Low | 08 (11.6) | 19 (28.4) | **0.044** | 0(0) | 2(66.7) |
| Moderate | 48 (69.6) | 32 (47.8) |  | 1(100) | 1(33.3) |
| Vigorous | 13 (18.8) | 16 (23.9) |  | 0(0) | 0(0) |
| ^a^Smoking, n (%) |  |  |  |  |  |
| Yes | 07 (10.1) | 23 (34.3) | **0.001** | 0(0) | 0(0) |
| No | 62 (89.9) | 44 (65.7) |  | 1(100) | 3(100) |
| ϮNDI, mean ± SD | 42.89 ± 4.52 | 44.1 ± 9.55 | 0.364 | 41.5 | 38 ±10.5 |
| VAS (0 - 10) | 6.22 ± 1.43 | 6.31 ± 1.34 | 0.73 | 5.75 | 6.0 ±1.0 |
| WHO 5 Index | 53.4 ± 14.48 | 49.59 ± 11.26 | 0.09 | 61.7 | 63.6 ±3.2 |

The values are presented as proportion and percentage (%) for categorical variable, indicated by ^a^Chi-square. Student t test was used for continuous variable and expressed as mean and standard deviation. SD – Standard deviation, DTC – Dynamic taping with conventional physiotherapy, STC – Sham taping with conventional physiotherapy, Mo – months. Ϯ – NDI expressed as 100 percent by doubling the score.

# Table 1b; Gender wise difference analysis of main effect and interaction effect for the PROM's comparison at baseline and post-intervention 4 weeks.

| **Covariate** | **Variable** | **Baseline mean (SD)** | **Post-intervention mean (SD)** | **F value (Within-Subject) time** | **F value (interaction)**  **Time*gender** |
| --- | --- | --- | --- | --- | --- |
| Gender (male, female) | NDI percent | 43.47 ± (7.4) | 33.2 ± (9.4) | 176.3  (p < 0.001) | 3.137  (p = 0.079) |
|  | VAS (0 to 10) | 6.25 ± (1.38) | 4.25 ± (1.23) | 245.5  (p < 0.001) | 0.339  (p < 0.67) |
|  | WHO 5 Index | 51.53 ± (13.1) | 54.67 ± (12.8) | 195.3  (p < 0.001) | 0.645  (p < 0.47) |
| **Post-hoc Bonferroni pairwise comparison estimates** | | | | | |
| **Gender**  (baseline versus post-intervention) | | **Mean difference (95% CI)** | | **Standard error** | **p value** |
| NDI score in percent | | 2.45 (-0.219, 5.135) | | 1.35 | 0.072 |
| VAS (0 to 10) | | 0.095 (-0.274, 0.463) | | 0.18 | 0.61 |
| WHO 5 Index | | -2.259 (-8.36, -0.157) | | 2.01 | 0.42 |

# Table 1c; Occupation wise difference analysis of main effect and interaction effect for the PROM's comparison at baseline and post-intervention 4 weeks.

| **Covariate** | **Variable** | **Baseline mean (SD)** | **Post-intervention mean (SD)** | **F value (Within-Subject) time** | **F value (interaction)**  **Time*gender** |
| --- | --- | --- | --- | --- | --- |
| Occupation (Clerical/student, business, professionals, skilled labor) | NDI percent | 43.41 ± (7.51) | 33.4 ± (9.54) | 221.6  (p < 0.001) | 0.67  (p = 0.56) |
|  | VAS (0 to 10) | 6.24 ± (1.37) | 4.29 ± (1.24) | 277.6  (p < 0.001) | 0.186  (p < 0.90) |
|  | WHO 5 Index | 51.82 ± (13.0) | 54.87 ± (12.7) | 79.77  (p < 0.001) | 0.169  (p < 0.97) |
| **Post-hoc Bonferroni pairwise comparison estimates** | | | | | |
| **Occupation** (baseline versus post-intervention) | | **Mean difference (95% CI)** | | **Standard error** | **p value** |
| NDI score in percent | | 10.18 (8.84, 11.53) | | 0.68 | 0.002 |
| VAS (0 to 10) | | 01.96 (1.73, 2.20) | | 0.11 | 0.000 |
| WHO 5 Index | | -3.033 (-3.71, -2.36) | | 0.34 | 0.000 |

# Table 1d; Level of physical activity wise difference analysis of main effect and interaction effect for the PROM's comparison at baseline and post-intervention 4 weeks.

| **Covariate** | **Variable** | **Baseline mean (SD)** | **Post-intervention mean (SD)** | **F value (Within-Subject) time** | **F value (interaction)**  **Time*gender** |
| --- | --- | --- | --- | --- | --- |
| Level of physical activity (low, moderate, vigorous) | NDI percent | 43.41 ± (7.51) | 33.41 ± (9.54) | 185.20  (p < 0.001) | 0.325  (p = 0.72) |
|  | VAS (0 to 10) | 6.24 ± (1.37) | 4.29 ± (1.24) | 206.6  (p < 0.001) | 4.11  (p < 0.018) |
|  | WHO 5 Index | 51.82 ± (13.2) | 54.87 ± (12.7) | 70.68  (p < 0.001) | 0.06  (p < 0.95) |
| **Post-hoc Bonferroni pairwise comparison estimates** | | | | | |
| **Level of physical activity** (baseline versus post-intervention) | | **Mean difference (95% CI)** | | **Standard error** | **p value** |
| NDI score in percent | | 10.13 (8.66, 11.60) | | 0.74 | 0.000 |
| VAS (0 to 10) | | 1.79 (1.55, 2.04) | | 0.125 | 0.000 |
| WHO 5 Index | | -3.103 (-3.83, -2.37) | | 0.36 | 0.000 |

***The post-hoc Turkey test*** *showed no significant difference between the low, moderate, and vigorous activity groups (NDI outcome measure:* Low versus moderate; mean diff 2.02, p 0.44. Low versus vigorous; mean diff 2.10, p 0.56. Moderate versus vigorous; mean diff 0.08, p 0.99. *VAS outcome measure:* Low versus moderate; mean diff 0.36, p 0.98. Low versus vigorous; mean diff 0.10, p 0.93. Moderate versus vigorous; mean diff 0.067, p 0.95. *WHO5I outcome measure:* Low versus moderate; mean diff – 1.58, p 0.83. Low versus vigorous; mean diff 0.17, p 0.99. Moderate versus vigorous; mean diff 1.74, p 0.80.)

# Table 1e; Smoking habit (smoker versus non-smokers) wise difference analysis of main effect and interaction effect for the PROM's comparison at baseline and post-intervention 4 weeks.

| **Covariate** | **Variable** | **Baseline mean (SD)** | **Post-intervention mean (SD)** | **F value (Within-Subject) time** | **F value (interaction)**  **Time*gender** |
| --- | --- | --- | --- | --- | --- |
| Smoking habits (Smokers versus non-smokers) | NDI percent | 43.41 ± (7.51) | 33.41 ± (9.54) | 143.39  (p < 0.001) | 0.62  (p = 0.43) |
|  | VAS (0 to 10) | 6.24 ± (1.37) | 4.29 ± (1.24) | 174.07  (p < 0.001) | 3.16  (p < 0.078) |
|  | WHO 5 Index | 51.82 ± (13.2) | 54.87 ± (12.7) | 50.14  (p < 0.001) | 1.008  (p 0.30) |
| **Post-hoc Bonferroni pairwise comparison estimates** | | | | | |
| **Smoking habits** (baseline versus post-intervention) | | **Mean difference (95% CI)** | | **Standard error** | **p value** |
| NDI score in percent | | 9.63 (8.05, 11.22) | | 0.81 | 0.000 |
| VAS (0 to 10) | | 1.81 (1.54, 2.08) | | 0.137 | 0.000 |
| WHO 5 Index | | -2.814 (-3.59, -2.03) | | 0.36 | 0.000 |

**Table 1 : Patient Reported Outcome measures (PROM) comparison at Baseline and Post- intervention 4 weeks by time and interaction of time including dropouts (Intention to Treat) (n = 140)**

| **Variable** | **Group** | **Baseline mean (SD)** | **Post-intervention mean (SD)** | **F value (Within-Subject) time** | **F value (interaction)**  **Time*gender** |
| --- | --- | --- | --- | --- | --- |
| NDI percent | STC (n = 70) | 43.8 ± (9.6) | 37.37 ± (9.74) | 289.2  (p < 0.001) | 36.9  (p < 0.001) |
|  | DTC (n = 70) | 43.02 ± (4.59) | 29.45 ± (7.53) |  |  |
| VAS (0 to 10) | STC (n = 70) | 6.28 ± (1.32) | 5.05 ± (1.11) | 413.17  (p<0.001) | 56.55  (p<0.001) |
|  | DTC (n = 70) | 6.20 ± (1.43) | 3.53 ± (0.82) |  |  |
| WHO 5 Index | STC (n = 70) | 50.2 ± (11.4) | 52.15 ± (10.6) | 94.42  (p<0.001) | 12.12  (p<0.001) |
|  | DTC (n = 70) | 53.45 ± (14.3) | 57.6 ± (14.1) |  |  |
